# Interfaces and Transport Phenomena in Materials Under Extreme Conditions

**DOI:** 10.3390/ma19091910

**Published:** 2026-05-06

**Authors:** Yanxi Chen, Run Hu, Haidong Wang

**Affiliations:** 1 Key Laboratory for Thermal Science and Power Engineering of Ministry of Education, Department of Engineering Mechanics, Tsinghua University, Beijing 100084, China; 2 School of Energy and Power Engineering, Huazhong University of Science and Technology, Wuhan 430074, China

Under extreme conditions, including atomic scale, ultra-fast processes, and ultra-high power density, the continuous-medium hypothesis and local equilibrium approximation of classical transport theory no longer hold, with interface effects governing system behavior. Understanding and precisely controlling interfacial behavior and transport phenomena under extreme conditions not only constitute a research frontier in materials science but also serve as a critical foundation for technological breakthroughs in semiconductor chips, high-energy lasers, aerospace, and other engineering fields. This Special Issue (SI) collects five original contributions reporting the latest theoretical, experimental, and simulation-derived advances regarding extreme-condition interfaces and transport phenomena. In this Editorial, we review cutting-edge research from three key dimensions—nanoscale systems, femtosecond-scale ultra-fast thermal processes, and ultra-high power density in advanced chips—and summarize the papers in this SI.

With the continuous development of material fabrication techniques, devices’ characteristic dimensions have been scaled down to the nanometer and even sub-nanometer scales. As the specific surface area increases significantly, geometrical confinement and interface effects gradually govern the thermo-mass transport processes, which, in turn, determine the macroscopic properties of materials. In the field of experimental characterization, state-of-the-art in situ vibrational electron energy-loss spectroscopy (EELS), implemented within electron microscopy, has enabled the direct measurement of temperature fields and interfacial thermal resistance at sub-nanometer resolution, providing a key methodology for unraveling the dynamics of interfacial phonon transport [1]. In the domain of numerical simulation, emerging approaches such as first-principles calculations and deep-neural-network-aided molecular dynamics have been applied to accurately describe transport phenomena at the nanoscale [2,3]. Unique transport mechanisms emerging at the nanoscale, such as defect-enhanced thermal transport, have also become a research hotspot in this field [4].

Advances in ultrafast laser technology facilitate the study of thermal transport at the picosecond-to-femtosecond time scale. In ultra-fast processes, thermal carriers (electrons and phonons) do not reach thermal equilibrium, so non-equilibrium transport and multi-field coupling are key scientific issues. Nonadiabatic molecular dynamics (NAMD) is a new method for simulating ultra-fast carrier dynamics. The surface-hopping scheme and multiple k-point first-principles NAMD have been used to simulate thermal-carrier excitation [5]. Measurements of interfacial thermal transport via the pump–probe thermos-reflectance technique have confirmed that volume-confined hyperbolic phonon polariton (HPhP) modes transfer energy across solid interfaces much faster than phonon–phonon thermal conduction [6]. Combining ultrafast temporal resolution and nanoscale spatial resolution has become a key approach for studying non-equilibrium phonon transport.

In applications such as laser ablation and high-power electronic devices, heat fluxes often reach the order of GW/m^2^, and internal temperature gradients in materials can reach hundreds of K/μm. Under such extreme conditions, the nonlocal effects of thermal transport and the multiscale coupling of power devices are the focus of modeling. One of the latest computational methods, namely, the nonlocal-heat-transport-theory-informed neural network, can capture dynamic transport behavior that is beyond the scope of classical models [7]. To address the ultra-high heat fluxes in devices, the electron and phonon transport mechanisms at the interface between room-temperature liquid metals and solid materials were analyzed, reducing interfacial thermal resistance [8]. Two-phase water-cooled micro heat exchangers designed based on the distribution manifold and hierarchical microchannel copper have also been studied, driving breakthroughs in the thermal management of high-power electronic chips [9].

Focusing on the theme of interfaces and transport phenomena in materials under extreme conditions, this SI collects five research papers featuring cutting-edge theoretical results, experimental techniques, and computational methods in this field, serving as a bridge for academic exchange and technological innovation in the field while providing useful guidance for relevant engineering practices. The contributions of this collection are summarized below.

For high-precision optoelectronic devices, fabricating upconversion (UC) nanofilms with precise thickness and facile patternability is crucial. Physical vapor deposition (PVD) can be used to accurately control film thickness but usually results in poor crystal structure and weak fluorescence. Zeng et al. [10] proposed an integrated Ni-UC nanofilm design that can enhance fluorescence by combining plasmonic nickel with surface plasmon resonance effects. This integrated design compensates for the shortcomings of PVD technology, highlighting integrated Ni-UC nanofilms’ application potential in advanced optoelectronics and sensing technologies.

Silicon carbide (SiC) is a wide-bandgap semiconductor with excellent thermal and mechanical properties, making it ideal for high-power, -frequency, and -temperature electronic applications. High-purity, large-area SiC semiconductors are typically fabricated on substrates via chemical vapor deposition (CVD). However, growing SiC on silicon substrates remains technically challenging because of the high defect density caused by lattice mismatch between silicon and SiC. Milenov et al. [11] investigated the CVD growth of SiC on silicon substrates with different orientations, exploring the feasibility of synthesizing intermediate thin SiC films at temperatures of up to 1150 °C—temperatures compatible with silicon-based technologies. This work provides guidance useful for the integration of SiC thin-film fabrication technology with silicon-based technologies.

Epoxy nanocomposites are widely used in various industrial fields, such as the automotive industry and aerospace, due to their inexpensiveness, excellent mechanical properties, and designability. However, their insufficient hardness, toughness, and thermal conductivity limit their application as friction materials. Tong et al. [12] fabricated epoxy nanocomposites containing both MoS_2_ nanosheets and aligned multi-walled carbon nanotubes by applying an external direct current (DC) electric field. This significantly enhanced the mechanical and tribological properties of the composites, further expanding their engineering applications.

Ferrovalley (FV) materials are an emerging class of 2D materials integrating ferromagnetic and valley characteristics. They have attracted a great deal of attention due to their potential to advance information processing and enhance data storage capacity. Janus structures form stable layers by combining halogens or sulfur with transition metals, and such innovative configurations hold promise as candidates for 2D FV materials. Chang et al. [13] employed first-principles calculations combined with ab initio molecular dynamics and Monte Carlo simulations to systematically investigate key properties of Janus TiTeCl monolayers, including ferromagnetism, in-plane magnetic anisotropy, valley polarization, and the quantum anomalous Hall effect. The authors identified their prospects for application as 2D FV materials in the field of advanced valleytronics.

Two-dimensional nanomaterials hold great promise as high-performance thermoelectric devices. However, their smallness and significant variability between samples hamper multi-parameter measurements. The T-type method and its evolved version, the H-type method, can be used to accurately measure multiple thermoelectric parameters of the same sample using a single system. Nevertheless, these methods require the use of DC voltage to heat a sample for Seebeck coefficient measurement, and the heating voltage is often much larger than the thermoelectric potential, impairing measurement accuracy. Zheng and Zhao et al. [14] proposed a measurement method combining laser heating with an H-type device. By introducing a temperature difference across the sample via laser heating, this method effectively eliminates the adverse effect of heating voltage on measurements while simplifying the structure of the device, providing a powerful tool for the precise characterization of the thermal and electrical properties of 2D nanomaterials.

In summary, research on interfaces and transport phenomena in materials under extreme conditions is a frontier of materials science and deeply coupled with engineering practices. The integrated paradigm of “materials innovation–interface engineering–multiscale simulation–intelligent design” has become a core pathway for advanced power device packaging and thermal management, and advances in machine learning and artificial intelligence (AI) have greatly accelerated the prediction of key interfacial transport properties [15]. Progress in regard to experimental and computational methods for investigating interfacial behaviors and transport phenomena under extreme conditions provides in-depth insights into physical mechanisms and lays a foundation for integration with AI. In addition, at more extreme scales, classical transport theories are being updated by new frameworks, such as nonlinear transport theory in non-centrosymmetric systems [16] and molecular-scale interfacial ordering based on intermolecular bonding interactions [17], keeping this field at the theoretical forefront. Future research on interfaces and transport phenomena in materials under extreme conditions will feature multiscale and multi-physics coupling, deep AI integration, and theoretical innovation. These endeavors will address new challenges in fields such as high-power electronic devices, high-energy laser systems, and high-speed optical communications and play an increasingly important role in engineering.
